# Recent Advances in Preventing and Managing Postoperative Delirium

**DOI:** 10.12688/f1000research.16780.1

**Published:** 2019-05-01

**Authors:** Phillip Vlisides, Michael Avidan

**Affiliations:** 1 Department of Anesthesiology, University of Michigan Medical School, Ann Arbor, Michigan, USA; 2 Center for Consciousness Science, University of Michigan Medical School,, Ann Arbor, MI, USA; 3 Department of Anesthesiology, Washington University School of Medicine, Saint Louis, Missouri, USA

**Keywords:** Anesthesia, Delirium, Cognitive Dysfunction, Cognitive Reserve, Neurocognitive, Neurophysiology, Postoperative, Surgery

## Abstract

Postoperative delirium is a common and harrowing complication in older surgical patients. Those with cognitive impairment or dementia are at especially high risk for developing postoperative delirium; ominously, it is hypothesized that delirium can accelerate cognitive decline and the onset of dementia, or worsen the severity of dementia. Awareness of delirium has grown in recent years as various medical societies have launched initiatives to prevent postoperative delirium and alleviate its impact. Unfortunately, delirium pathophysiology is not well understood and this likely contributes to the current state of low-quality evidence that informs perioperative guidelines. Along these lines, recent prevention trials involving ketamine and dexmedetomidine have demonstrated inconsistent findings. Non-pharmacologic multicomponent initiatives, such as the Hospital Elder Life Program, have consistently reduced delirium incidence and burden across various hospital settings. However, a substantial portion of delirium occurrences are still not prevented, and effective prevention and management strategies are needed to complement such multicomponent non-pharmacologic therapies. In this narrative review, we examine the current understanding of delirium neurobiology and summarize the present state of prevention and management efforts.

## Introduction

Delirium is an enigmatic clinical syndrome characterized by an acute and typically reversible failure of our brain’s basic cognitive and attentional functions. Delirium can be associated with alterations in level of consciousness and is characterized by a fluctuating course. Those with delirium are often either agitated (hyperactive type) or lethargic (hypoactive type) or alternate between these motor subtypes (mixed type). Delirium occurs commonly in older adults, especially when there is pre-existing neurocognitive impairment and also following an insult such as an infection or a trauma. With many vulnerable older adults requiring surgery, postoperative delirium specifically is a growing public health concern, occurring with an incidence of 20 to 50% in those older than 60 after major surgery ^1, 2^. When patients become delirious, this is often the most distressing element of the perioperative experience, both for them and for their family members. Furthermore, postoperative delirium is associated with increased mortality ^3^, cognitive and functional decline ^4– 6^, increased hospital length of stay ^7^, and substantial annual health-care costs ^8^. Despite the grave nature of delirium and its associated burdens, foundational problems have tempered the pace of clinical and scientific progress. Most fundamentally, the pathophysiology of delirium phenomenology ^9^ remains incompletely understood. Although it is appropriate that diagnosis of a clinical syndrome is informed by bedside observations, this ideally should be accompanied by a clear understanding of the underlying pathophysiology. Additionally, delirium screening tools (for example, the Confusion Assessment Method for the Intensive Care Unit and the Delirium Observation Screening scale) used in routine clinical practice demonstrate low sensitivity (about 30%) compared with expert-based delirium identification (that is, psychiatrist, geriatrician, or neurologist performing chart reviews and completing similar delirium screening assessments) ^10^. Furthermore, diagnostic disagreement may be common among such experts ^10^. In fact, diagnostic discrepancy even occurs with different *Diagnostic and Statistical Manual of Mental Disorders* (DSM) editions. In an examination of a pooled dataset of prospective studies investigating delirium, strict DSM-5 criteria identified only 30% of delirium cases diagnosed via DSM-IV criteria ^11^. Although guidelines have been published for the prevention of postoperative delirium ^12– 14^, they are often supported by low-quality evidence ^12, 15, 16^. Furthermore, implementation efforts may be limited by the required administrative support, resources, and health-care staff education ^17, 18^. Thus, with an incomplete pathophysiologic understanding, a deficient diagnostic toolbox, and limited guideline evidence and implementation capacity, prevention and management of delirium are inherently challenging.

Multiple steps can be taken to improve clinical understanding and management of delirium. First, elucidating the neurobiologic mechanisms of delirium will advance understanding of the syndrome. These efforts could help produce targeted therapeutic strategies that address and alleviate intrinsic pathophysiologic perturbations. Second, improvement in current guideline implementation and adherence may still reduce delirium incidence and improve related outcomes ^19^. In this narrative review, we examine the current understanding of delirium pathophysiology and summarize what is known regarding prevention and management efforts. Future directions are briefly discussed with a focus on improving diagnostic and management strategies.

## Delirium pathogenesis: current understanding

Delirium classification and diagnosis currently rely on phenotypic descriptions of altered brain states (for example, inattention and disorganized thinking) rather than a neurobiologically informed framework. By comparison, perioperative cardiovascular perturbations (for example, wall motion abnormalities and tamponade) can be diagnosed at the bedside with currently available technology and diagnostic acumen. No analogous, standard neurophysiologic evaluation process exists for diagnosing or evaluating the severity of altered postoperative brain states. This deficit in pathophysiologic understanding likely contributes to the current state of ineffective prevention and management. This has been reinforced by recent large pharmacologic trials that have failed to demonstrate reductions in postoperative delirium risk despite promising preliminary data ^2, 20^. Additionally, a systematic review in 2016 demonstrated that then-current pharmacologic treatment strategies were ineffective for reducing delirium duration or severity ^21^. Our hope is that such informative trials will galvanize investigative efforts to better understand the pathophysiology of delirium and related brain states ^22^.

The pathophysiologic framework of delirium has evolved over recent years with advances in neurocognitive research. From a systems neuroscience perspective, neurotransmitter imbalances—particularly involving dopamine and acetylcholine—have been implicated in delirium pathogenesis ^23^. Sleep disruption and polypharmacy may contribute to such neurotransmitter alterations (reviewed in Watson *et al*. ^24^). Neuroinflammation may also play a role as specific neuroinflammatory protein signatures track with postoperative delirium ^25, 26^. The net effect of these perturbations may manifest as network-level alterations in information processing. In fact, using 21-channel electroencephalographic (EEG) data, van Dellen *et al*. demonstrated reduced functional connectivity, altered directionality of information flow, and network topology changes during delirious episodes in cardiac surgery patients ^27^. This group published subsequent data comparing EEG measures of hypoactive delirium with non-delirious controls and those recovering from anesthesia in the immediate postoperative setting ^28^. Hypoactive delirium was distinguished from these other states by network topology features, as measures of network integration in the alpha band were reduced. Network science may help shape our understanding of brain state transitions perioperatively (reviewed in Lee and Mashour ^29^) and this could apply to delirium as well as to other altered brain states. Shafi *et al*. have proposed a model by which transcranial magnetic stimulation could be used to assess connectivity and neuroplasticity in real time ^22^, hypothesizing that reduced baseline connectivity and plasticity contribute to delirium risk. This neurophysiologic line of investigation ultimately may produce bedside tools for objective risk stratification and diagnosis of altered brain states, based on the underlying neurobiology. Preliminary work, based on frontal-parietal oscillatory patterns, has already shown promise in identifying delirium with high reliability against reference DSM-IV–based criteria ^30^.

## Recent prevention strategies

Despite the knowledge gaps in delirium pathogenesis, delirium may still be preventable with targeted, multicomponent interventions ^31^. Given the harmful nature of delirium and the apparent failure of currently used drugs (for example, haloperidol) for prophylaxis and treatment ^21^, prevention efforts have expanded through recent investigation of novel pharmacologic and non-pharmacologic strategies.

### Pharmacologic

Ketamine has been found to reduce postoperative inflammation ^32^, improve perioperative pain outcomes ^33^, and reduce excitotoxicity in laboratory models ^34^. Results from a small trial also demonstrated decreased occurrence of delirium and decreased incidence of delayed neurocognitive recovery in cardiac surgery patients who received intraoperative ketamine compared with placebo ^35, 36^. With this background and rationale, an international team of investigators conducted the PODCAST (Prevention of Delirium and Complications Associated with Surgical Treatments) trial, hypothesizing that a sub-anesthetic, intraoperative dose of ketamine would reduce postoperative delirium ^37^. In this trial, ketamine had no statistically significant effect on delirium incidence (ketamine groups: 19.45%, placebo group: 19.82%, absolute difference 0.36%, 95% confidence interval [CI] −6.07 to 7.38; *P* = 0.92), delirium severity, or delirium recurrence ^2^. Instead, dose-dependent increases were reported for postoperative hallucinations (18% in the placebo group, 20% in the 0.5 mg/kg ketamine group, and 28% in the 1.0 mg/kg ketamine group; *P* = 0.01) and nightmares (8% in the placebo group, 12% in the 0.5 mg/kg ketamine group, and 15% in the 1.0 mg/kg ketamine group; *P* = 0.03). These findings align with previously known psychoactive side effects of ketamine ^33, 38, 39^. Thus, intraoperative ketamine probably does not prevent delirium; rather, ketamine may increase the risk of adverse perioperative psychoactive experiences.

Dexmedetomidine has also been tested in large randomized trials in relation to postoperative delirium. A 2014 meta-analysis examined dexmedetomidine use across 14 trials involving cardiac surgery and intensive care unit (ICU) patients ^40^. In these trials, dexmedetomidine was investigated as a sedation agent, primarily for mechanically ventilated patients, compared with gamma-aminobutyric acid–based sedative-hypnotics (for example, propofol and midazolam). In this context, dexmedetomidine use was associated with reduction in the composite outcome of delirium, agitation, and confusion (relative risk [RR] 0.68, 95% CI 0.49 to 0.96; *P* = 0.03). Similar findings were presented in a meta-analysis by Duan *et al*. ^41^. Delirium incidence was significantly lower in surgical populations receiving dexmedetomidine (odds ratio [OR] 0.35, 95% CI 0.24 to 0.51; I ^2^ = 53%). However, the two largest studies in the meta-analysis may provide a nuanced perspective. The largest trial was performed by Su *et al*., who conducted a 700-patient, double-blinded, randomized controlled trial in which dexmedetomidine, administered in the ICU until the morning after major non-cardiac surgery, was associated with an impressive 13.4% absolute reduction (95% CI 8.1% to 18.7%) in postoperative delirium incidence ^42^. However, there are some concerns regarding the methodology of this trial ^43^. The majority of participants (nearly 60%) were consented by proxy postoperatively, and participants were not tested for delirium at the time of enrollment. Thus, the primary outcome was likely present in some patients in both groups prior to the intervention. Furthermore, the biological plausibility remains in question, as a robust delirium reduction was achieved with a small, sub-sedative dose rate (0.1 μg/kg per hour). Nonetheless, preliminary data demonstrate that low-dose-rate dexmedetomidine may improve perioperative sleep ^44– 46^, which has been postulated to mitigate delirium risk ^47^. A trial by Deiner *et al*. demonstrated no significant difference in delirium incidence between dexmedetomidine (12.2%) and placebo (11.4%) groups (RR 1.06, 95% CI 0.79 to 1.41; *P* = 0.77) ^20^. The study design was different, as dexmedetomidine was started intraoperatively and continued for only 2 hours postoperatively. Nonetheless, the trial was stopped early for futility, as dexmedetomidine did not appear to influence delirium risk or cognitive function 3 to 6 months after surgery. Of note, however, relevant confounders (for example, anesthetic and analgesic consumption) were not comprehensively reported and this contributed to trial limitations. Overall, dexmedetomidine may mitigate delirium risk in mechanically ventilated, critically ill patients; however, its prophylactic use in the intraoperative and immediate postoperative setting, particularly for non-cardiac surgery patients, remains controversial ^43, 48^. Large multicenter trials, with preoperative delirium testing, rigorous delirium assessment, and multiple treatment arms (for dose comparisons), are warranted to refine the evidence regarding the role of dexmedetomidine in preventing or treating delirium.

Apart from ketamine and dexmedetomidine, other drugs have shown some promise as prophylactic agents in both cardiac and non-cardiac surgery. These include acetaminophen, ramelteon, gabapentin, statins, clonidine, and melatonin ^49, 50^. Recently, a small, industry-funded, single-center trial demonstrated that intravenous acetaminophen every 6 hours for 48 hours after cardiac surgery was associated with an impressive 18% (95% CI −32 to −5%; *P* = 0.01) absolute risk reduction in delirium incidence compared with placebo ^51^. However, this result, as noted by the investigators ^51^, should be viewed as hypothesis-generating only. The biological plausibility of acetaminophen decreasing delirium incidence, especially to such a large extent, is questionable. Therefore, even with this encouraging finding, the probability that acetaminophen is effective at preventing delirium should still be regarded as low ^52^. A common misunderstanding is that *P* values provide direct information regarding the probability of the truth or falsity of hypotheses ^53^. The *P* value, if inappropriately used for (null) hypothesis testing, substantially overstates the evidence against the null hypothesis ^54^. The fragility index (which suffers from the same limitations as *P* values) has been proposed to assess the robustness of positive results in clinical trials ^55^. The fragility index ^55^ calculation for this trial ^51^ indicates that if just two patients in the acetaminophen group were “converted” to having delirium, the results would lose statistical significance at an arbitrary *P* value of less than 0.05. Another major constraint of this trial was that the control group received placebo ^56^ rather than oral or rectal acetaminophen, which often is standard practice after cardiac surgery. In order to adopt a new expensive treatment, like intravenous acetaminophen, it would be necessary to show that it was superior to inexpensive alternatives, like the generic oral formulation of the same drug. Given these important limitations, the results of this trial should be tested for reproducibility in a large, multicenter trial, as the investigators themselves have recommended ^51^. Atypical antipsychotic agents, such as haloperidol and quetiapine, have not shown benefit in preventing or treating delirium ^57^. Similarly, steroids, which have non-specific anti-inflammatory properties, have not been effective at preventing postoperative delirium ^58^. Pending more compelling evidence, no pharmacologic agent currently can be recommended for prophylaxis of postoperative delirium ^49^.

### Depth of anesthesia

**Figure 1.**
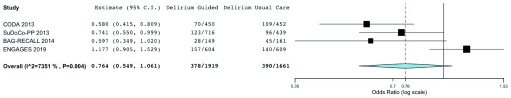
Meta-analysis summarizing four trials in which the intervention group received electroencephalogram-guided anesthesia. This analysis was conducted by using OpenMetaAnalyst ^65^ and was based on a binary, random effects, Hartung–Knapp–Sidik–Jonkman model ^66, 67^. The I ^2^ = 74%, tau ^2^ = 0.08, Q(df = 3) = 13.234, and heterogeneity *P* value = 0.004. The estimated odds ratio for delirium with intervention (electroencephalogram-guided [reduction in] anesthesia) = 0.764 (95% confidence interval 0.549 to 1.061, *P* = 0.108). BAG-RECALL, Bispectral Index or Anesthesia Gas to Reduce Explicit Recall; C.I., confidence interval; CODA, Cognitive Dysfunction after Anesthesia; ENGAGES, Electroencephalography Guidance of Anesthesia to Alleviate Geriatric Syndromes; SuDoCo-PP, Surgery Depth of Anaesthesia and Cognitive Outcome per-protocol.

Within the last decade, a growing body of evidence has implicated anesthetic depth as a possible contributor to postoperative delirium. The Cognitive Dysfunction after Anesthesia (CODA) trial was published in 2013. Of 1000 patients who were planned for randomization, 921 older non-cardiac surgery patients (≥60 years of age) were randomly assigned to bispectral index (BIS)-guided anesthesia versus routine care ^59^. General anesthesia was achieved with ether-derived inhaled agents or with propofol intravenous anesthesia. Postoperative delirium, which was assessed in 902 patients, was reduced by 8.6% (95% CI 3.4 to 13.7) (relative reduction of 35%, 95% CI 16 to 51%) in the BIS-guided group, and cognitive dysfunction was also less common in the guided group 3 months after surgery. Of note, however, delirium was a secondary outcome of the trial, delirium was assessed only once daily, information on missing delirium data was not reported, delirium assessment training was not discussed, and protocol deviations were not reported. The same year, Radtke *et al*. reported findings from the Surgery Depth of Anaesthesia and Cognitive Outcome (SuDoCo) trial, which enrolled 1277 older non-cardiac surgery patients ^60^. Notably, the investigators specified *a priori* that 1600 patients would be enrolled (ISRCTN Register: 36437985), but the study was stopped early because of shortage of funds. General anesthesia was either with ether-derived inhaled agents or with propofol intravenous anesthesia. Interestingly, mean BIS values were almost identical (~39) in the BIS-guided and blinded groups. Delirium incidence was reported for 90.4% (1155 patients) and was significantly reduced in the BIS-guided group (16.5% versus 21.4%, absolute reduction 4.9%, 95% CI 0.3 to 9.4%; *P* = 0.036) ^60^. However, in the BIS-blinded group, clinicians deviated from the study protocol and unblinded themselves to BIS values for 141 patients at some point during surgery. By conducting the analysis with these patients in the BIS-guided group (that is, per-protocol approach), the association between BIS monitoring and delirium is not statistically significant (17.2% versus 21.9%, absolute reduction 4.7%, 95% CI −0.1 to 9.4%; *P* = 0.053). Observational data also demonstrate an association between intraoperative EEG suppression and postoperative delirium risk, even after adjustment for relevant confounders ^61, 62^. Collectively, these studies suggest that deep anesthesia—which is marked by EEG suppression—may causally contribute to postoperative delirium. Alternatively, the excessive presence of EEG suppression may reflect underlying neurologic vulnerability, indicating a higher inherent risk of delirium. The Electroencephalography Guidance of Anesthesia to Alleviate Geriatric Syndromes (ENGAGES) trial (ClinicalTrials.gov Identifier: NCT02241655) addressed this question by randomly assigning 1232 surgical patients to EEG-guided anesthesia—with a focus on avoiding EEG suppression—versus usual, EEG-blinded care ^63, 64^. Postoperative delirium occurred in 157 (26.0%) out of 604 patients in the EEG-guided group compared with the 140 (23.0%) out of 609 in the usual care group (absolute difference 3.0%, 95% CI −2.0 to 8.0; *P* = 0.22). The EEG-guided group had 46% (95% CI 16 to 76%) less EEG suppression time and 14% (95% CI 12 to 16%) less volatile anesthetic exposure. The findings suggest that EEG-guided anesthesia probably does not reduce postoperative delirium occurrence substantially in older surgical patients, even if EEG suppression time during surgery is decreased. The trial had specific methodological strengths, including structured delirium assessment training, fidelity checks for protocol compliance, and validated chart review methods to complement in-person delirium interviews. The ENGAGES trial also had several limitations, including the following: (i) single-center design, potentially limiting generalizability; (ii) lack of objective diagnostic criteria or biomarkers for delirium, which is a common consideration for all studies focusing on delirium; (iii) the potential for missed delirium occurrences given that delirium is a fluctuating disorder and could be missed with interval or insufficient assessments; and (iv) the potentially limited applicability to general anesthesia based on intravenous anesthetic agents. The results from these trials are illustrated meta-analytically in Figure 1.

Findings from the ENGAGES trial are consistent with those of systematic reviews of hip fracture surgery studies that have found no association between anesthetic technique (that is, general versus neuraxial anesthesia) and postoperative delirium risk ^68– 70^. Similar findings were demonstrated in the STRIDE **(**Strategy to Reduce the Incidence of Postoperative Delirium in Elderly Patients) trial ^71^, which randomly assigned patients undergoing surgery for hip fracture (n = 200) to light versus heavy sedation during spinal anesthesia. Overall, there was no significant difference in delirium incidence in the light sedation group (34/100, 34%) compared with the heavy sedation group (39/100, 39%; absolute reduction 5.0%, 95% CI −8.3 to 18.3%; *P =* 0.46). Thus, both the ENGAGES trial and data from the hip fracture surgery literature do not support current recommendations to use EEG-guided anesthesia for patients at risk in order to prevent postoperative delirium ^12^. This conclusion may be refined after the findings of the ENGAGES-Canada trial (ClinicalTrials.gov Identifier: NCT02692300) and Balanced Anesthesia Trial ^72^ are published.

### Behavioral and multicomponent interventions

One of the most consistently effective delirium prevention strategies involves a multicomponent intervention that targets modifiable risk factors. The Hospital Elder Life Program (HELP), founded by Sharon K. Inouye *et al*., is a multidisciplinary program designed to prevent cognitive and functional decline in older hospitalized patients, and the focus is on delirium ^73^. HELP services include cognitive orientation, social support, sleep protocol implementation, assistance with nutrition and mobilization, and education for health-care staff. HELP has expanded to over 200 sites worldwide, and positive outcomes have been reproduced across several hospital settings and locations. A recent meta-analysis involving 14 studies demonstrated significant reductions in delirium incidence (OR 0.47, 95% CI 0.37 to 0.59; I ^2^ = 28%), risk of falls (OR 0.58, 95% CI 0.35 to 0.95; I ^2^ = 0%), and health-care costs ($16,000 USD per person-year) ^31^. Despite the paucity of effective delirium prevention strategies, HELP stands as a consistent, reproducible intervention for preventing delirium in high-risk patients.

Cognitive prehabilitation is also being studied as an approach for strengthening cognitive reserve in surgical patients ^74^. So-called “brain training” efforts have been hypothesized to curtail the risk of postoperative delirium and cognitive impairment. Computerized cognitive training exercises have demonstrated cognitive benefit in non-surgical patients across a wide variety of clinical settings ^75^. However, modest gains are generally observed in the short term, and training appears to require direct supervision, over several hours, and spaced out over multiple weeks to avoid cognitive fatigue ^75, 76^. In fact, preliminary data demonstrate that such training programs are unlikely to be feasible for many older patients ^77^. Time commitment and preoperative anxiety served as barriers to training adherence, and those randomly assigned to training were more likely to withdraw from the study. Although larger-scale trials are ongoing (ClinicalTrials.gov Identifier: NCT02230605 ^74^), cognitive prehabilitation may not be feasible for many older patients prior to surgery.

## Clinical management

Medical associations such as the UK’s National Institute for Health and Care Excellence, the European Society of Anaesthesiology, and the American Geriatrics Society offer evidence-based guidelines for postoperative delirium management ^12, 13, 78^. Initial steps focus on identifying and treating precipitating etiologies. In hospitalized patients, iatrogenic causes include infection, polypharmacy, fluid and electrolyte disturbances, and organ failure (with associated physiologic perturbations). Concurrent with treating the underlying medical condition, supportive efforts can be implemented to mitigate delirium severity. Non-pharmacologic interventions, such as delirium education programs for medical staff, have led to reductions in delirium duration, hospital length of stay, and mortality ^79^. Such programs can also improve delirium recognition and disposition and are associated with reductions in point prevalence ^80^. Pharmacologic interventions for treating active delirium have been studied for many years, although most studies have not found candidate drugs to be effective. Neufeld *et al*. recently published a systematic review to examine antipsychotic medication treatment for delirium ^21^. The authors reviewed 19 studies, which included various typical and atypical antipsychotics across diverse hospital settings, and found that antipsychotics demonstrated no significant effects on delirium incidence, duration, or severity, or on hospital length of stay. In fact, a subsequent clinical trial by Agar *et al*. demonstrated improved survival, reduced delirium severity scores, and fewer extrapyramidal effects in the placebo group compared with risperidone and haloperidol arms in palliative care patients ^81^. Thus, current guidelines recommend only pharmacologic treatment for select scenarios, such as severe agitation (that is, posing harm to self or others or both) and alcohol or benzodiazepine withdrawal ^12, 13, 78^.

Lastly, the lack of delirium guideline implementation may also impede delirium prevention and care, especially in the ICU. A recent prospective mixed-methods study by Balas *et al*. ^18^ examined barriers to guideline dissemination and implementation across various ICU settings. Participants reported that (1) knowledge deficits and (2) low confidence with using delirium screening tools, particularly as time elapsed after initial training and education, served as barriers for delirium guideline implementation. These findings align with similar studies involving medical wards, where a staff educational program reduced delirium incidence and related complications, including mortality and hospital length of stay ^79^. Thus, consistent educational and training efforts may help prevent delirium and associated deleterious outcomes.

## Conclusions and future directions

Delirium is a distressing syndrome for older surgical patients and their families, and the societal consequences of delirium are likely to escalate with a growing older surgical population. Advancing our pathophysiologic understanding of delirium is likely to inform better screening and diagnostic strategies. Neurophysiologic investigation, shaped by a network science framework, may improve neurobiologic understanding of delirium mechanisms. Knowledge gaps in relation to pathophysiology may help explain why rigorous, large pharmacologic and non-pharmacologic trials for delirium prevention have generally been disappointing ^2, 20, 64^ and weigh against current guidelines ^13^. Non-pharmacologic, multicomponent interventions are not likely to increase the risk of harm and have repeatedly been shown to reduce the incidence and impact of delirium ^31^. With improving scientific and technological advances and the establishment of multidisciplinary neuroscience collaborations ^82, 83^, the time is ripe to improve delirium understanding and management.
